# Behavioral avoidance of contagious and non-contagious adults

**DOI:** 10.1371/journal.pone.0272726

**Published:** 2022-08-11

**Authors:** Vanessa LoBue, Emily Kim, Laura Marrone, Katy-Ann Blacker, Gretchen Van de Walle

**Affiliations:** Department of Psychology, Rutgers University, Newark, New Jersey, United States of America; St John’s University, UNITED KINGDOM

## Abstract

Evolutionary theories of disease avoidance propose that humans have a set of universal psychological processes to detect environmental cues indicative of infectious disease. These processes then initiate cognitive, emotional, and behavioral responses that function to limit contact with harmful pathogens. Here, we study the conditions under which people exhibit behavioral avoidance of others with a contagious illness or a physical injury (i.e., a broken leg), and the potential mechanisms that underlie this behavior. Across three studies, participants were given the option of sitting at one of two workstations previously occupied by two confederates, one of whom either showed visible symptoms of a cold (contagion condition), wore a lower-leg orthopedic boot and used crutches (broken leg condition), or showed no signs of illness or physical injury (control). We found strong evidence that adults explicitly avoid contact with individuals who show symptoms of a contagious illness. Further, we provide some evidence that adults also avoid individuals with a physical injury, but that this behavior might be driven by implicit, unconscious processes. The findings are discussed in terms of implications for the healthy avoidance of contagion, and the risk for potential stigmatization of non-contagious groups.

## Introduction

Evolutionary theories of disease avoidance propose that, because of the significant reproductive fitness costs associated with pathogen exposure, humans have evolved psychological mechanisms that facilitate the avoidance of potential exposure to disease. These psychological mechanisms are thought to detect cues in the environment that signal the presence of pathogens or infectious illness, and initiate cognitive and emotional responses, such as disgust, that lead to avoidance behavior [1–9].

Although these theories differ in their specifics, a prominent feature of virtually all evolutionary theories of disease avoidance is hypersensitivity to perceptual indicators of potential pathogen presence to avoid costly false negative errors [4, 10–13]. This highly conservative heuristic bias makes sense given that outward manifestations of diseases vary considerably. Failure to avoid an infected individual is a potentially lethal error, whereas erroneously avoiding a healthy individual carries relatively few costs. Thus, much of the evidence offered in support of these theories has focused on the overextension of attitudinal and emotional responses associated with infection to individuals who exhibit atypical features that are potentially, though not actually, consistent with contagious illness (e.g., facial disfigurement, skin conditions, disability, obesity).

It may appear self-evident that adults tend to avoid the threat of infectious disease, and indeed social stigmatization of individuals with illnesses such as HIV/AIDS, SARS, and Ebola is not uncommon [14, 15]. However, surprisingly few studies have investigated behavioral responses to the presence of contagious individuals. Instead, research on disease avoidance has primarily examined people’s *attitudes* and *beliefs* about both contagious and non-contagious individuals, with a special focus on the overgeneralization of beliefs and attitudes about individuals suffering from contagious illnesses to those suffering from non-contagious illnesses or conditions. Further, because evolved psychological processes are thought to be most active when people consider themselves to be particularly vulnerable to infection, research in this area has also focused on the responses of individuals who have been primed to think about parasitic transmission or who are especially sensitive to disease vulnerability.

For example, individuals who perceive themselves to be vulnerable to potential infection and whose awareness of the risks of pathogen transmission has been temporarily heightened through exposure to a series of disease-related images demonstrate greater antipathy toward obese individuals than those for whom pathogen concerns are less prominent [16]. Similarly, those who perceive themselves to be more likely to get sick have fewer friends with disabilities [17], and show strong associations between unpleasantness and disease for elderly individuals on an implicit association task [18]. These findings have been interpreted as evidence that evolved disease avoidance mechanisms are so robust that they overgeneralize to people with non-contagious conditions that nonetheless deviate from a prototypical healthy phenotype.

Although most of the work on psychological disease avoidance mechanisms focuses on overgeneralization to non-contagious conditions in people who perceive themselves to be particularly vulnerable to infection, a few studies have specifically examined attitudes and beliefs about contagious illnesses in healthy adults. Not surprisingly, adults believe that contagious illnesses are more likely to be transmitted to others than non-contagious illnesses and report a greater desire to avoid individuals with contagious illnesses than non-contagious illnesses [11]. Similarly, adults report being less willing to make physical contact (e.g., shaking hands, sitting next to individuals on a bus) with individuals who have a visible facial lesion than with individuals who appear to be physically healthy. Adults also express a greater level of discomfort with the idea of making physical contact (e.g., shaking hands) than nonphysical contact (e.g., speaking on the phone) with individuals who have contagious diseases, and interestingly, although to a lesser extent, they also extend this discomfort to individuals with non-standard morphologies such as limb amputation or obesity [19]. Finally, a recent study showed that adults demonstrate equal unwillingness to contact individuals with obesity and the flu [20].

Taken together, this research provides evidence that people report a desire to avoid illness, harbor negative attitudes toward sick people, and overgeneralize these attitudes towards individuals whose appearance is not, but could be, associated with infection. Importantly, in all these studies, participants were presented with vignettes or photographs and were asked to report their likely behavior (typically via questionnaire) rather than having to take any actual action. It remains relatively unknown whether such beliefs and desires to avoid both contagious and non-contagious individuals would also support *behavioral avoidance*. Indeed, it is possible that the responses measured in much of the existing research reflect psychological processes that are sensitive to information potentially associated with perceived pathogen presence, but which may not actually promote pathogen avoidance behavior [21]. Thus, the evaluation of behavioral responses to a range of cues that are associated or not associated with actual diseases should enable researchers to more precisely specify the processes that consistently support pathogen avoidance and its generalization.

A handful of studies examine adults’ actual behavior towards contagious and non-contagious individuals and the objects they touch. However, the results of these studies are somewhat mixed. For example, one study reported that adults are more willing to touch a bandage that had contacted a disgusting but non-infectious injury when compared to an equally disgusting but contagious injury, suggesting that people are more willing to touch objects that non-contagious people have touched over objects that contagious people have touched [22]. In contrast, another study reported that participants were less likely to make oral contact with objects touched by someone with influenza *and* by someone with a non-contagious facial birthmark when compared to a control condition [23], suggesting that people are equally likely to avoid contact with objects touched by contagious and non-contagious individuals with atypical physical features. However, while some research shows that adults avoid physical proximity to individuals who have a facial birthmark [24], or are handicapped [25, 26], another study demonstrated adults do not avoid proximity to someone who is physically handicapped unless they have an explicit excuse to do so [27], suggesting that avoidance of the physically disabled might be situation-specific.

Altogether, it is unclear from this work whether adults consistently avoid only people who are contagious, or whether they generalize avoidance behavior to non-contagious individuals with a physical atypicality, as evolutionary theories of pathogen avoidance would predict. One possible explanation for these inconsistencies is that the *mechanisms* that underlie avoidance of both contagious and non-contagious individuals varies. According to some evolutionary theories of illness avoidance, the underlying mechanism driving pathogen avoidance is disgust [6]. Indeed, there is a large literature linking disgust sensitivity to general avoidance behavior [28–30], to avoidance of contaminated objects [31–33], and to avoidance of objects contaminated by illnesses like the flu or common cold [34]. However, one study examining individuals’ subjective feelings about contagious individuals and individuals with non-contagious injuries demonstrated that while people’s responses to contagious individuals are related to disgust, their responses to non-contagious injuries are driven by different emotional mechanisms, like empathy [22]. This suggests that avoidance of contagious versus non-contagious individuals might be driven by different mechanisms.

Other studies suggest that avoidance of non-contagious individuals might be driven by implicit biases related to stigmatization. For example, one study examining adults’ behavioral responses to non-contagious individuals in a virtual reality platform reported that avoidance responses were the greatest for stigmatized groups like people with HIV when compared to non-stigmatized groups like individuals with cancer [35]. Further, there is large literature suggesting that while adults typically report positive attitudes and feelings towards individuals belonging to stigmatized groups (i.e., individuals with physical disabilities, individuals of different racial/ethnic backgrounds, the elderly) when tested using direct self-report measures, they show a significant bias *against* these individuals when tested with more indirect measures, such as response time, EEG/ERP, or the implicit associations test (IAT) [36]. Thus, it is possible that while avoidance responses to individuals with contagious illnesses might be driven by explicit and conscious mechanisms, avoidance of non-contagious individuals might be driven by more implicit, non-conscious mechanisms.

In the current research, we examined adults’ behavioral avoidance of surfaces that had been touched by a contagious and non-contagious individual, and the potential underlying mechanisms driving these avoidance responses in a naturalistic set of experiments. Across three studies, participants were given the option of sitting in one of two workstations previously occupied by two confederates, one of which either showed visible symptoms of a cold (contagion condition), wore a lower leg boot and used crutches (broken leg condition), or showed no signs of illness or physical injury (control). Our primary question was whether participants would only avoid sitting in the location previously occupied by the contagious individual, or whether they would also avoid sitting in the location previously occupied by the confederate with a broken leg. Our secondary question was whether we would find evidence that avoidance of the contagious and non-contagious individuals was driven by the same explicit mechanism, or whether avoidance of the non-contagious individual is more likely to be implicit.

## Experiment 1

All stimuli and data can be accessed on Databrary. The design, analyses, and hypotheses for Experiment 3 were all preregistered on AsPredicted #15264. Experiments 1 and 2 were not preregistered, as they were conducted before preregistration was widely available. However, sample sizes across all studies were determined before data was collected and analyzed and were based on previous research using a similar paradigm with a similar outcome variable. All studies were conducted before the onset of the COVID-19 pandemic in 2020.

### Method

#### Participants

All participants were recruited from the Rutgers-Newark Psychology Department participant pool and received partial credit towards their general requirements for psychology courses. The Rutgers University Institutional Review Board approved all procedures, and all participants provided informed consent. We used a behavioral paradigm similar to that of Snyder et al. [27] with a dichotomous outcome; these authors did not report an effect size, but they tested 25 adults per condition. Thus, here we were slightly more conservative and tested 30 adults per condition.

Ninety undergraduate students (30 per condition) from the Rutgers University-Newark campus participated in Experiment 1, (45 female; *M*_age_ = 21 years; 3 participants did not report their age). An additional 19 participants were tested but excluded from analysis because of experimenter error (2), failure on the manipulation check (9), and/or the memory check (7).

We did not collect data on participants’ race/ethnicity, but all participants across all three studies were recruited from the Rutgers-Newark participant pool. In the Fall of 2020, 765 total students were enrolled in the participant pool, and of those students, 122 (15.9%) identified as Black or African American, 81 (10.6%) as Asian or Asian American, 93 (12.2%) as White or European American, 255 (33.3%) as Latino or Hispanic, 83 (10.8%) as Middle Eastern or North African, 41 (5.4%) as South Asian or Asian Indian, 39 (5.1%) as Multiracial, 2 (0.3%) as American Indian or Alaska Native, 1 (0.1%) as Native Hawaiian or Pacific Islander, 21 (2.7%) identified as some other race, and 27 (3.5%) declined to respond.

#### Stimuli

Six ~7-minute videos served as the stimuli. Each video depicted a room with the same two female confederates sitting at separate desks, facing away from each other, each typing on a laptop computer. In the *contagion* condition, one of the two confederates (target) periodically blows her nose into a tissue and coughs while typing. In the *broken leg* condition, the same confederate is wearing a leg boot and a set of crutches is propped against the side of her desk. In the *control* condition, the same confederate has no visible ailment or physical injury. Near the end of the ~7-minute video, the target confederate closes her laptop and leaves the room, followed by the second confederate. In the broken leg video, the confederate in the leg boot uses the crutches as she leaves the room. There were two videos for each condition, one with the target confederate sitting at the desk on the left, and one with the target confederate sitting at the desk on the right.

#### Apparatus

Two lab spaces were used to conduct the study. The first space was arranged as an office, with a desk and computer, a couch, and a small flat screen monitor sitting on the desk providing a “live feed” into the second lab space. The second lab space was across the hall from the office space, and contained two small tables, each with a chair and laptop, facing away from each other. This is the same space depicted in the videos.

#### Procedure

Participants were randomly assigned to one of the three video conditions (contagious, broken leg, control), counterbalanced across participant gender, and the side of the room where the target confederate was sitting on the video. The experimenter began playing the assigned video on the monitor just before the participant entered the room at the designated appointment time. Upon arrival, the experimenter asked the participant to sit on the couch, which was located with a direct view of the monitor, and explained that the study involved taking a survey on a laptop in a second lab space. Gesturing towards the monitor, the experimenter then explained that the previous study was running late, and once the two women depicted on the screen were finished filling out their surveys, the current study could begin. The participant was then given paperwork to fill out while the video continued to play.

After the two women on the video appeared to exit the lab, the experimenter told the participant that they could begin the study and led the participant out of the office and across the hall into the lab space. Again, the lab was arranged exactly as it had been depicted on the video, with two desks facing away from each other, each with a laptop. Upon entering, the experimenter told the participant to “sit anywhere” and then went into a back room momentarily, leaving the participant to choose which of the two desks to sit at. The experimenter then returned and instructed the participant to fill out a survey (the *Perceived Vulnerability to Disease* scale, described below) on the laptop. After finishing the survey, participants were asked: 1) if they believed that the livestream video was live (manipulation check), and 2) if they remembered anything specific about the videos, and which side of the room the target confederate was sitting (memory check). They were then debriefed. The experimenter recorded where the participants chose to sit and the participants’ responses to the memory and manipulation check questions.

#### Measures

The main dependent measure of interest was where the participants chose to sit—in the seat previously occupied by the target or control confederate. They also filled out the *Perceived Vulnerability to Disease (PVD)* scale, which is a validated, 15-item self-report measure of perceived vulnerability to illness and emotional discomfort in the presence of illness [37]. The PVD is comprised of two subscales which measure germ aversion (e.g., “It really bothers me when people sneeze without covering their mouths”) and perceived infectability (e.g., “If an illness is ‘going around’, I will get it”). All responses are measured on a Likert scale from 1 (strongly disagree) to 7 (strongly agree).

### Results and discussion

The main variable of interest was a binary measure of whether participants sat in the seat previously occupied by the target confederate. Thus, in the following analyses, to test for differences from chance we used binomial tests; and to test for differences between the video conditions, we used chi-square analyses, with confidence intervals and effect sizes (*φ*). Post-hoc comparisons between individual video conditions were done using additional, Bonferroni-corrected chi-square analyses. Finally, we ran a series of logistic regressions on each condition with avoidance as the dependent variable and the germ aversion and perceived infectability subscales of the PVD entered as predictors, to see if individual differences in sensitivity to illness transmission was related to avoidance in each condition.

In Experiment 1, 24/30 participants (80%) avoided sitting where the contagious confederate previously sat, compared to 21/30 (70%) for the broken leg confederate, and 11/30 (37%) for the target confederate in the control condition. A chi-square analysis revealed significant differences between the video conditions, *χ*^*2*^ (2, 90) = 13.14, *p* < .001, *φ* = .382, 95% CI [0.52, 0.72]. Post-hoc comparisons (with Bonferroni corrected critical *p* = 0.017, for 3 comparisons) revealed a significant difference between the control and contagious condition, *χ*^*2*^ (1, 60) = 11.59, *p* < 0.001; however, there was also a significant difference between the control and broken-leg conditions, *χ*^*2*^ (1, 60) = 6.70, *p* = .010, and no differences between contagion and broken leg conditions, *χ*^*2*^ (1, 60) = 0.80, *p* = 0.371. A follow-up series of binomial tests confirmed that the participants in the contagion (*p* < 0.001) and the broken leg (*p* = 0.043) conditions avoided the seat occupied by the target confederate more often than would be predicted by chance, but participants in the control condition did not (*p* = 0.200). Logistic regressions revealed that neither subscale of the PVD predicted avoidance behavior (*p*’s > 0.05).

The results of Experiment 1 confirm that adults do avoid sitting in a location previously occupied by a potentially contagious adult. That avoidance was not predicted by either subscale of the PVD is not surprising; adults’ explicit knowledge of germs and contagion would very likely have swamped individual differences in perceived susceptibility to illness or aversion to germs. Participants also avoided sitting in place of a non-contagious adult with a broken leg, consistent with the idea that adults might overgeneralize avoidance behavioral responses associated with contagious individuals to individuals who exhibit a physical injury.

One possible explanation for these findings is that participants made a conscious, explicit decision to avoid the desk previously occupied by the confederate with the broken leg because they thought it might be reserved for individuals with a physical handicap or out of concern that the previous, injured participant might return and need access to the desk. A related possibility is that participants in this condition chose to avoid the desk out of a conscious, explicit desire to avoid contagion. An alternate explanation is that because most adults know that individuals with a broken leg do not have a contagious illness, avoidance in the broken leg condition was implicit, or unconscious. The fact that avoidance in the broken leg condition was not predicted by scores on either subscale of the PVD suggests that any implicit biases in the broken leg condition may not have been associated with concerns about contagion, although this possibility cannot be ruled out. However, as mentioned above, previous research suggests that while adults typically report positive attitudes and feelings towards individuals with physical disabilities when tested using self-report measures, they nonetheless show significant evidence of bias *against* individuals with physical disabilities, the elderly, and individuals of different racial/ethnic backgrounds when tested with more indirect measures [36].

In Experiment 2, we attempted to determine whether avoidance in Experiment 1 was driven by explicit, articulable concerns with contagion and/or disability or by more implicit influences. We presented adults with short excerpts of the videos that participants saw in Experiment 1, but instead of asking them to make a behavioral choice between the two seats, we instead asked them to indicate which desk they *would* sit at, and later to explain the reason for their decision. We predicted that if in Experiment 1, avoidance of both the contagious confederate and the confederate with a broken leg was explicit and intentional, participants in Experiment 2 should still choose to avoid the desk at which both confederates once sat and provide a specific explanation for their choice. In contrast, if behavior in the two conditions in Experiment 1 was driven by different mechanisms, participants might still avoid the contaminated desk, but they may not explicitly avoid the desk previously occupied by the confederate with a broken leg.

## Experiment 2

### Method

#### Participants

Ninety undergraduate students (30 in each condition) from the Rutgers University-Newark campus participated in Experiment 2, (45 female; *M*_age_ = 21 years). An additional participant was tested but excluded from analyses because of failure to complete the survey. All participants were recruited from the Psychology Department participant pool and received partial credit towards their general requirements for psychology courses. The Rutgers University Institutional Review Board approved all procedures, and all participants provided informed consent.

#### Stimuli

A 2-minute excerpt from each of the videos from Experiment 1 served as the stimuli for Experiment 2.

#### Procedure

Participants were randomly assigned to one of the three video conditions (contagious, broken leg, control), counterbalanced across participant gender, and the side of the room in which the target confederate was sitting on the video. Each participant responded to a set of questions presented on a desktop computer (Qualtrics, Provo UT) in a lab space in the presence of an experimenter. First, participants were prompted to watch the 2-minute video with the volume on. After the video, they were prompted to answer several questions. First, they were asked: If you had to enter the room you saw depicted in the video to complete a survey after the 2 participants left, where would you choose to sit? The question appeared below an image of the room with the two desks empty, and with the left and right sides clearly labeled. They were then asked to explain why they would choose to sit there. Finally, they were asked whether they remembered which of the two women in the video was the target (had a cold, a broken leg, or for the control condition, wore a black dress and black shoes) and to indicate whether she was sitting at the left or the right desk. Finally, they were asked whether they thought they could get sick by sitting in the same seat as the target confederate, whether they thought they could get a broken leg by sitting in the same seat as the target confederate, and finally, why, or why not for each.

### Results and discussion

**Fig 1:**
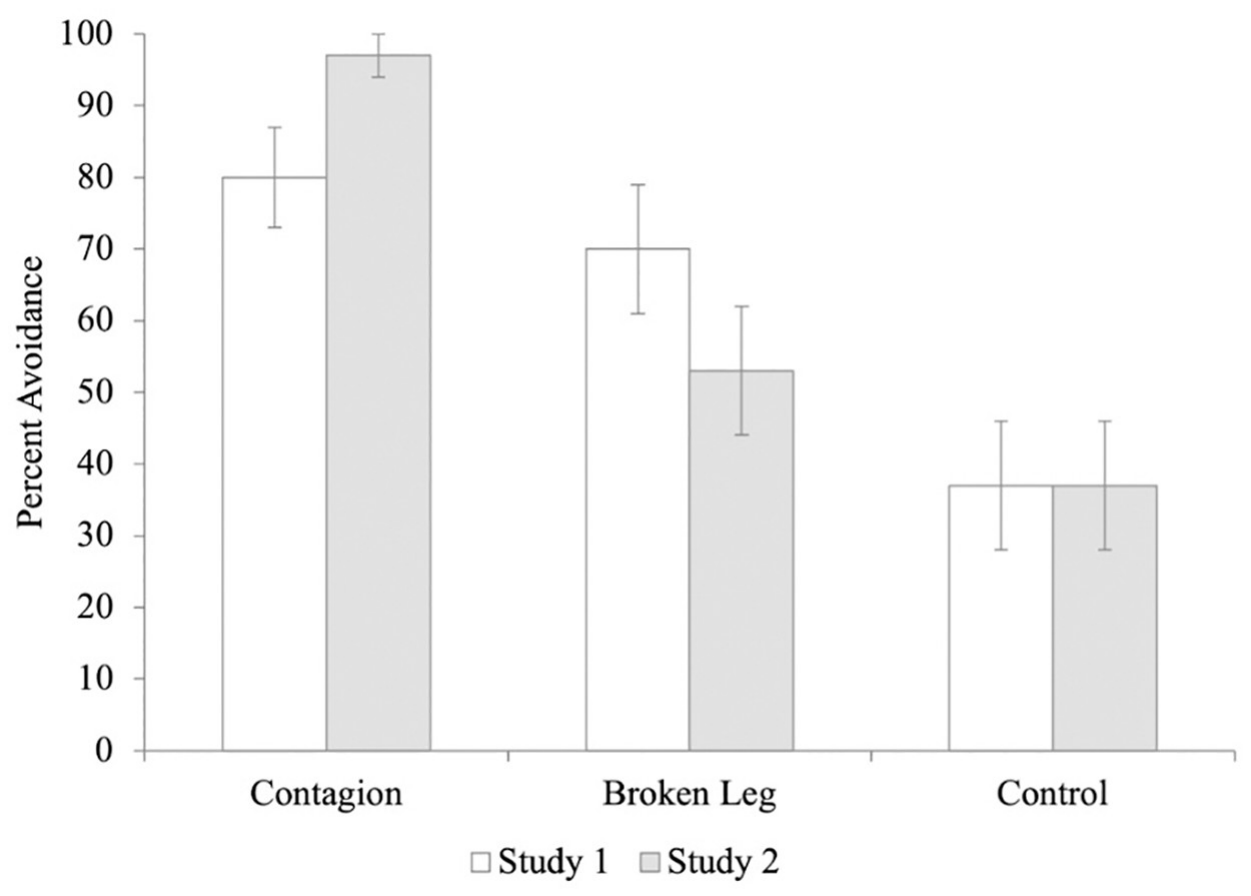
Percentage of adults who avoided the target confederate in Experiments 1 and 2.

In Experiment 2, 29/30 participants (97%) said that they would avoid sitting in the seat of the contagious confederate, compared to only 16/30 (53%) for the broken leg confederate and 11/30 (37%) for the confederate in the control video (Fig 1). As in Experiment 1, a chi-square analysis revealed significant differences between the video conditions, *χ*^*2*^ (2, 90) = 24.49, *p* < .001, *φ* = .522, 95% CI [0.52, 0.72]. However, inconsistent with Experiment 1, post-hoc comparisons (with Bonferroni corrected critical *p* < 0.017, for 3 comparisons) revealed that there was a significant difference between the control and contagious condition, *χ*^*2*^ (1, 60) = 24.30, *p* < 0.001, and between the contagious and broken leg conditions, *χ*^*2*^ (1, 60) = 15.02, *p* < 0.001; the control and broken-leg condition did not differ, *χ*^*2*^ (1, 60) = 1.68, *p* = 0.194. A follow-up series of binomial tests confirmed that the participants in the contagion (*p* < 0.001) condition avoided the target confederate significantly above chance, but not in the broken leg (*p* = 0.856) or control conditions (*p* = 0.200). Again, logistic regressions revealed that scores on neither subscale of the PVD predicted avoidance in any of the video conditions.

When asked why they chose their seat, 27/30 (90%) of participants in the contagious condition said something specific about avoiding illness (e.g., “I don’t want to get sick”, “The other person was coughing a lot”). However, in the broken leg condition, only 2/30 (7%) participants said something about the seat potentially being reserved for the person with the broken leg (“So the other injured person could sit comfortabl[y]”, “To give the person with the broken leg more personal space so they can move around without me being in their way”). None of the participants in the broken leg condition said that they could get sick by sitting in place of the confederate with the broken leg, whereas all the participants in the contagious condition said that they could possibly get sick by sitting in place of the contagious confederate.

These results suggest that the choice to avoid sitting in a potentially contagious person’s seat is both intentional and explicit, as evidenced by 97% of the participants in Experiment 2 reporting that they would avoid sitting in the sick confederate’s seat, and 90% explicitly mentioning illness or contagion as the reason for their choice. In contrast, participants in Experiment 2 were at chance when choosing between seats in the broken leg condition. Thus, when making a behavioral choice, like in Experiment 1, adults avoided sitting in a confederate’s seat if they had a broken leg, but they showed no such avoidance if asked to make an explicit, verbal judgement about where they would sit. Taken together, these results suggest that adults do avoid contact with a potentially contagious person’s seat and generalize avoidance behavior to individuals who exhibit a physical injury. However, while avoidance of a visibly sick individual is a conscious and explicit choice, avoidance of non-sick individuals who exhibit a physical injury might be more implicit or unconscious.

An alternative (though we think unlikely) explanation is that participants in both Experiments 1 and 2 indeed wished to avoid the individual with the broken leg on the basis of explicit, conscious desire to avoid contagion. On this account, participants in Experiment 1 avoided the individual with the broken leg since they had to make an actual behavioral choice that carried a real possibility of contracting an illness. In contrast, since participants in Experiment 2 only had to make a verbal response, incurring no actual behavioral consequences, they may have simply chosen to provide the more socially desirable response in the broken leg condition.

Experiment 3 attempts to evaluate the influence of making an explicit, verbal response on participants’ behavioral choice to occupy or avoid a workstation previously occupied by a visibly contagious individual or an individual with a broken leg. In Experiment 3, three conditions were identical to those in Experiment 1. Participants saw one of three videos of two confederates working at desks with laptops. One of the confederates appeared to have a cold, wore a leg boot and used crutches, or showed no symptoms of illness and wore no leg boot. When participants were presented with a choice between sitting at one of the two desks, the experimenter said, “sit anywhere” and left the room, leaving the participant unobserved while making their behavioral choice. Experiment 3 also had three conditions in which the videos were identical, but instead of instructing participants to “sit anywhere,” the experimenter explicitly asked the participants, “where do you want to sit?” and then remained in the room while the participant took their seat. Thus, in all conditions in Experiment 3, participants had to make a behavioral choice—to sit down and fill out a survey at one of the two laptops. However, they only had to provide an explicit, verbal response in one set of conditions. Thus, the first three conditions were a direct replication of Experiment 1; the second three conditions were like Experiment 2 in that, participants had to make an explicit verbal choice about where to sit, but similar to Experiment 1 in that they then had to follow through by actually sitting at the chosen desk.

If avoidance in the contagion and broken leg conditions in Experiment 1 were driven by the same explicit mechanism, we expected that participants would be equally likely to avoid the target seat/laptop regardless of the experimenter’s prompt (i.e., “sit anywhere” versus “where do you want to sit”). However, if avoidance in the broken leg condition in Experiment 1 was more implicit or unconscious, we expected that the two broken leg conditions would differ as a function of the experimenter’s prompt, with significant avoidance when participants were told to “sit anywhere,” (as in Experiment 1) but no avoidance when asked to make an explicit, verbal choice about where to sit (as in Experiment 2).

## Experiment 3

### Method

As in Experiments 1 and 2, we aimed to collect data from 30 participants per condition (180 participants total). However, data collection was interrupted by the onset of the COVID-19 pandemic. Given that this study is directly related to illness avoidance, we felt that any data collected before the pandemic might be qualitatively different than data collected after the onset of the pandemic. In fact, data for this study would have been impossible to collect after the onset of the pandemic, both because of social distancing restrictions and because participants would be highly unlikely to believe that a contagious individual would be permitted to enter a lab without any remediation. As a result, instead of collecting data from 30 participants per condition, we were only able to collect data from 155 participants (between 24 and 28 per condition) before the COVID-related shutdown. The results will be discussed with this limitation in mind.

#### Participants

One-hundred fifty-five undergraduate students from the Rutgers University-Newark campus participated in Experiment 3, (86 female; *M*_age_ = 21 years). An additional 127 participants were tested but eliminated from the study because of experimenter error (5), missing videos (22), camera malfunctions (3), and failure on the manipulation check (64) and/or memory check (49). The Rutgers University Institutional Review Board approved all procedures, and all participants provided informed consent.

#### Stimuli

The same videos from Experiment 1 were used for Experiment 3.

#### Procedure

Participants were randomly assigned to one of the three video conditions (contagious, broken leg, control) and one of two prompt conditions (verbal or behavioral), counterbalanced across participant gender, and the side of the room in which the target confederate was sitting on the video. The same method was used as Experiment 1 except that when entering the lab space, participants assigned to the behavioral condition were asked to “sit anywhere” (replication of Experiment 1) and participants assigned to the verbal condition were asked “where do you want to sit?” Additionally, once the participant had finished, the experimenter asked, “Why did you choose the seat you are sitting in?” before asking the manipulation and memory check questions.

### Results and discussion

**Fig 2:**
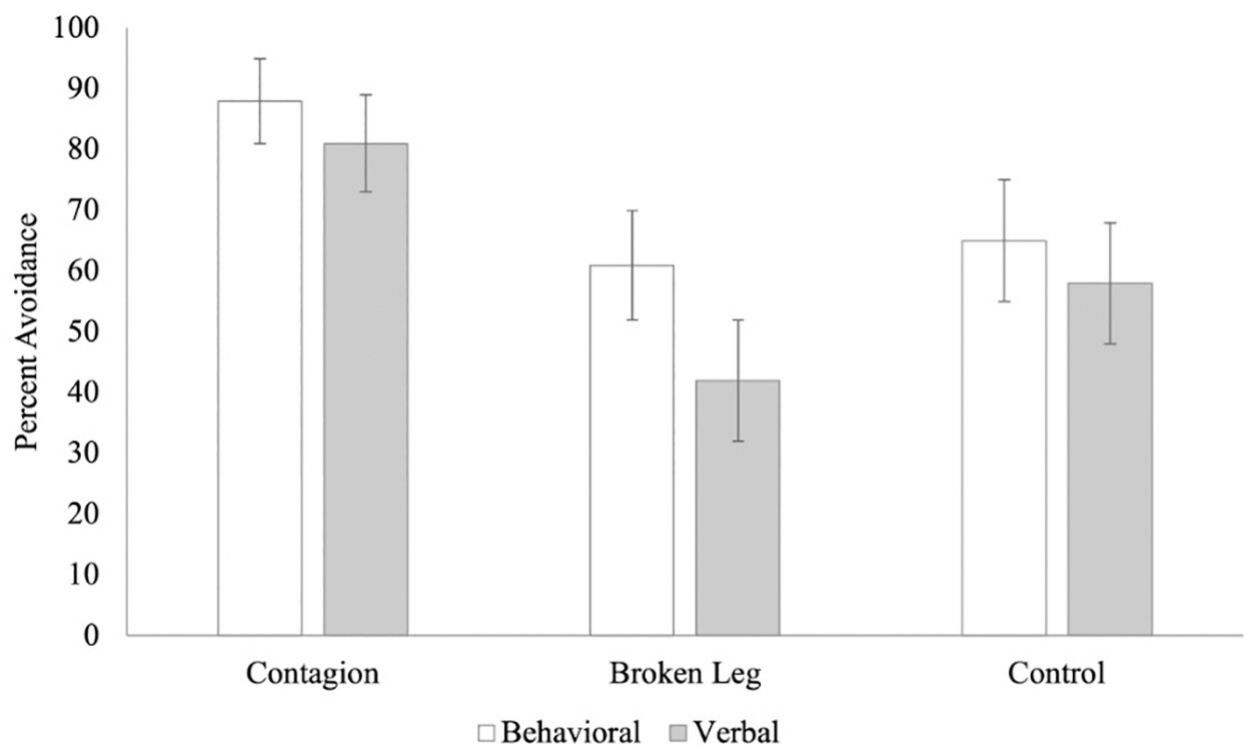
The Percentage of adults who avoided the target confederate in Experiment 3.

In the behavioral condition, 22/25 participants (88%) avoided sitting in the seat of the contagious confederate, compared to 17/28 (61%) for the broken leg confederate and 17/26 (65%) for the confederate in the control video. A chi-square analysis revealed a difference between the video conditions that was not significant, only trending in the predicted direction, *χ*^*2*^ (2, 79) = 5.33, *p* = .069, *φ* = .260, 95% CI [0.49, 0.72]. In the verbal condition, 21/26 participants (81%) avoided sitting in the seat of the contagious confederate, compared to 10/24 (42%) for the broken leg confederate and 15/26 (58%) for the confederate in the control video. A chi-square analysis revealed a significant difference between the video conditions, *χ*^*2*^ (2, 76) = 8.12, *p* = .017, *φ* = .327, 95% CI [0.61, 0.81]. A follow-up series of binomial tests confirmed that the adults avoided only the contagious confederate significantly above chance, in both behavioral (*p* < 0.001) and verbal (*p* = 0.002) conditions (all other *p’*s > 0.01) (see Fig 2). When asked why they chose their seats, 13/25 (52%) in the behavioral contagious condition and 13/26 (50%) in the verbal contagious condition mentioned something contagion-related in their explanation. No one in the broken leg condition mentioned the confederate’s leg. Again, logistic regressions revealed that scores on two subscales of the PVD did not significantly predict avoidance in any of the video conditions (*p*’s > 0.05).

To avoid inflating our error rate, instead of running follow-up post-hoc tests comparing the contagion and broken leg conditions to the control condition, we instead compared the behavioral and verbal broken leg conditions to each other, since this comparison provides the most direct test of our hypothesis. Although the trend was in the predicted direction, with 42% of participants avoiding the confederate’s workspace with the broken leg in the verbal condition compared to 61% in the behavioral condition, the chi-square was not significant, *χ*^*2*^ (1, 52) = 1.88, *p* = .171. Given that we did not reach our target sample size, we did a post-hoc power analysis to examine whether we had enough power to detect a significant effect in this particular comparison. First, we did this by calculating Cohen’s *d* for the comparison, based on the means and standard deviations of the two groups (verbal, *M* = .42, *SD* = .50; behavioral, *M* = .61, *SD* = .50), which resulted in a *d* of 0.38. We then used G*Power to calculate power to detect a significant effect based on this *d* and based on our sample sizes in each group (*N* = 24, verbal; *N* = 28, behavioral). On these factors, we only had 29% power to detect a small to medium-sized effect.

It is noteworthy that while very few participants failed the memory and manipulation checks in Experiment 1 (N = 16), a large number of participants did so in Experiment 3 (N = 97). Because this study requires a set-up where participants must believe that they are watching a live video feed, it is possible that the experimenters differed in their ability to convince participants that the set-up was real. Different experimenters ran the two experiments; the experimenter for Experiment 1 had significantly more experience conducting research, which could have led to the difference in memory/manipulation check failures between studies. However, to ensure that the large number of dropped participants in Experiment 3 did not significantly affect our results, we ran the chi-square analyses and binomial tests again with the full sample, including the 97 participants who were initially eliminated. The results were mostly the same, except that the chi-square analyses for the verbal condition were not significant, *χ*^*2*^ (2, 116) = 2.68, *p* = .262, *φ* = .152, 95% CI [0.45, 0.63], and the binomial test for the contagious confederate in the verbal condition only approached significance, (*p* = 0.088). This is not surprising, as the dropped participants either did not notice the contagious confederate or did not believe that they were watching a live feed.

Overall, the results of Experiment 3 confirm that participants consistently avoid individuals who show signs of contagious illness. However, participants did not avoid an individual with a broken leg in either the behavioral or the verbal condition. The difference between the two broken leg conditions was in the predicted direction—with greater avoidance in the behavioral than in the verbal condition—but it did not reach statistical significance despite a medium effect size, likely because of lack of power.

## General discussion

Over recent decades, several theorists have suggested that humans evolved not only physiological but also psychological defenses that protect against potentially lethal infection. However, until now, researchers have largely neglected the investigation of behavioral responses to actual disease cues, for both contagious and non-contagious individuals. This is important, as the examination of behavioral outcomes would enable a much higher degree of precision and detail in theories of actual pathogen avoidance and could shed considerable light on the mechanisms that underlie these processes. Further, behavioral avoidance of contagious individuals is what is most relevant to whether people in fact get sick and spread illnesses to others, making this an important outcome to measure to further refine these theories. Here, we examined adults’ behavioral avoidance of confederates who showed symptoms of a contagious illness and a physical injury, providing a test of both whether adults avoid contact with contagious individuals and whether they generalize behavioral avoidance of contagious individuals to non-contagious individuals.

Across three studies, we found consistent and robust evidence of contamination avoidance—participants reliably avoided contact with a potentially contagious individual’s workstation in all three experiments and when asked to explain this choice made clear reference to contamination and/or the potential transmission of illness. Generalization of behavioral avoidance to an individual with a physical injury, in contrast, was mixed. In Experiment 1, adults avoided sitting in place of a confederate who had a broken leg. When asked in Experiment 2 to provide a verbal response instead of a behavioral one, participants showed no evidence of avoidance. In Experiment 3, we attempted to replicate the findings of Experiment 1 and to further test whether asking participants to first verbally report where they wanted to sit would affect their later behavioral responses. We found that participants were more likely to avoid sitting in the place of someone with a broken leg when making an exclusively behavioral choice than when having to first make a verbal choice, but this difference was not significant despite a small to medium effect size, and overall, there was no significant avoidance of the confederate with the broken leg in Experiment 3.

Interestingly, across all three experiments, we found no relation between avoidance responses and scores on the Perceived Vulnerability to Disease (PVD) scale. It is likely that even individuals who see themselves as relatively invulnerable to disease and germs nonetheless avoid clearly contaminated objects when given a choice, as did over 80% of participants across our experiments. The lack of association between PVD scores and avoidance of an individual with a broken leg is consistent with the possibility that behavioral avoidance, though more implicit, was not in fact associated with disease-related biases. However, this interpretation should be viewed as speculative, as it is also possible that we had too little variability in our behavioral responses to detect individual differences based on the PVD with our current sample size.

Indeed, an overall concern about the non-significant results in Experiment 3 was a lack of power, as we were not able to reach our recruitment goal of 30 participants per condition because of the onset of the COVID-19 pandemic. Despite a medium effect size of *d* = 0.39, we only had 29% power to detect this effect (68% power in Study 1, and 80% power in Study 2 according to post-hoc power analyses). Thus, it is likely that reduced power from not reaching our target sample of 30 per condition influenced our results. However, it is important to note that despite limited power, the results of the two contagion conditions were still significant, suggesting that while avoidance of contagious individuals is quite robust, avoidance of a non-contagious individual with a physical injury might be more variable or situation specific, consistent with previous research [27].

Further, while physical handicaps, obesity, and old age are all stigmatized groups within the United States [36], a broken leg may not be a particularly stigmatized physical condition that people seek to avoid. For example, it is possible that the temporary nature of a broken leg distinguishes this attribute from other physical conditions that might elicit more avoidance. This possibility could be investigated by systematically comparing temporary versus permanent physical features—for example, by comparing behavioral avoidance of an individual with a brace/cast (perceptually salient and distinctive, yet temporary) versus an artificial limb (perceptually similar to a natural limb, but indicative of a permanent condition), or an individual who is pregnant versus obese. Thus, our results might have underestimated the degree to which adults avoid individuals based on their physical features. Future research examining variability in adults’ avoidance based on different physical characteristics is needed to clarify this issue.

Despite the non-significant of results for the broken leg conditions in Experiment 3, this work provides some evidence that adults avoid contact with both contagious individuals and individuals with a physical injury in some contexts. Further, it provides some support for the idea that avoidance of contagious individuals is explicit and intentional, while avoidance of individuals with a physical injury might be more implicit. This is consistent with previous research on stigmatization. Indeed, as mentioned above, although adults generally report positive attitudes and feelings towards individuals with physical disabilities when tested using self-report measures, they often show significant evidence of bias against individuals with physical disabilities, the elderly, and individuals of different races/ethnicities when tested with indirect, implicit measures [36]. Further, these negative implicit attitudes can influence behavior, even if explicit attitudes are positive. In fact, researchers have suggested that implicit attitudes may *better* predict spontaneous behavior than explicit attitudes, which are more deliberative and easier to control [38]. This is consistent with what we found here across studies: Indeed, while participants avoided both the contagious confederate and the confederate with the broken leg when making a behavioral choice in Experiment 1, avoidance fell to chance in Experiment 2 for the broken leg condition when participants instead had to make a verbal choice. In Experiment 3 when we differentiated between behavioral choices that were or were not accompanied by a verbal response, there was a trend for less avoidance when a verbal response was required. Further, across all studies, individuals were able to clearly articulate that avoidance in the contagious conditions was explicitly aimed at avoiding illness transmission. There was no such clear articulation of targeted avoidance in the broken leg conditions in any of the studies. However, it is important to acknowledge that while we believe our findings suggest that avoidance of non-contagious individuals is implicit, given the non-significant results of Experiment 3, we cannot rule out alternative explanations, such as the possibility that in Studies 2 and 3, participants did not avoid the confederate with the broken leg because they were adhering to social desirability norms. Further research is needed to distinguish between these possibilities.

On a broader level, the positive implication of these findings is that if the tendency to avoid individuals with a physical injury is implicit, recognition of this bias can help people regulate and reduce it to avoid stigmatization (Dovidio et al., 2011). The negative implication is that these data were collected *before* the COVID-19 pandemic, and it is possible that the onset of the pandemic might inflate people’s tendency to avoid individuals with any physical atypicality, potentially increasing the incidence of stigmatization. Theoretically, evolved psychological processes are thought to be most active when people consider themselves to be particularly vulnerable to infection. Indeed, individuals who are in a heightened state of vulnerability to disease demonstrate greater antipathy toward the elderly, and toward individuals who are obese or have physical disabilities [16–18]. Very recent work has shown that both disgust sensitivity and perceived vulnerability to disease (as measured by the PVD) have increased since the onset of the pandemic [39], are associated with more anxiety about COVID-19, and are associated with engagement in more preventative behaviors like social distancing [40–43]. Thus, when we consider these findings in the context of the recent global pandemic, greater sensitivity to the possibility of getting sick might make avoidance of non-contagious individuals, like someone with a physical injury, more likely. Further research is needed to investigate this possibility.

It is important to point out both the strengths and limitations of the current research. First, using a behavioral and naturalistic paradigm helps us understand how people would actually behave in the real world when confronted with contagious and non-contagious individuals. However, behavioral paradigms also have weaknesses. For example, collecting behavioral data is more time-consuming than collecting self-reports, and unavoidable power issues constrained our ability to draw conclusions about the findings of our third experiment. Further, using a dichotomous outcome variable (although not uncommon in previous research [23, 27]) limits our ability to capture variability in participants’ behavioral responses, and to relate our behavioral responses to self-reports on the PVD. It is also important to acknowledge that while our findings are suggestive of different mechanisms for the avoidance of contagious and non-contagious individuals, we are not suggesting that these are the *only* mechanisms that can drive such avoidance behavior. Indeed, emotional responses like disgust could certainly underlie avoidance behavior of both contagious and non-contagious individuals depending on the circumstance [6, 44]. Likewise, cognitive mechanisms could drive avoidance as well. Thus, one question is how different mechanisms for avoidance behavior might function differentially based on context and culture, and how they might differ with development or age. This is an important consideration for future work in this domain.

In conclusion, the current work provides strong evidence that adults avoid contact with individuals who show symptoms of a contagious illness. Further, we provide some weaker evidence that adults also avoid contact with individuals with a physical injury. The findings have implications for the healthy avoidance of contagion, and the risk for potential stigmatization of non-contagious groups.
